# Development and validation of a nomogram for predicting in-hospital mortality of patients with cervical spine fractures without spinal cord injury

**DOI:** 10.1186/s40001-024-01655-4

**Published:** 2024-01-29

**Authors:** Zhibin Xing, Lingli Cai, Yuxuan Wu, Pengfei Shen, Xiaochen Fu, Yiwen Xu, Jing Wang

**Affiliations:** The First Affiliated Hospital of Jinan University, Guangzhou, China

**Keywords:** Cervical spine fracture, Mortality, Nomogram, Predictive model, Intensive care unit

## Abstract

**Background:**

The incidence of cervical spine fractures is increasing every day, causing a huge burden on society. This study aimed to develop and verify a nomogram to predict the in-hospital mortality of patients with cervical spine fractures without spinal cord injury. This could help clinicians understand the clinical outcome of such patients at an early stage and make appropriate decisions to improve their prognosis.

**Methods:**

This study included 394 patients with cervical spine fractures from the Medical Information Mart for Intensive Care III database, and 40 clinical indicators of each patient on the first day of admission to the intensive care unit were collected. The independent risk factors were screened using the Least Absolute Shrinkage and Selection Operator regression analysis method, a multi-factor logistic regression model was established, nomograms were developed, and internal validation was performed. A receiver operating characteristic (ROC) curve was drawn, and the area under the ROC curve (AUC), net reclassification improvement (NRI), and integrated discrimination improvement (IDI) were calculated to evaluate the discrimination of the model. Moreover, the consistency between the actual probability and predicted probability was reflected using the calibration curve and Hosmer–Lemeshow (HL) test. A decision curve analysis (DCA) was performed, and the nomogram was compared with the scoring system commonly used in clinical practice to evaluate the clinical net benefit.

**Results:**

The nomogram indicators included the systolic blood pressure, oxygen saturation, respiratory rate, bicarbonate, and simplified acute physiology score (SAPS) II. The results showed that our model had satisfactory predictive ability, with an AUC of 0.907 (95% confidence interval [CI] = 0.853–0.961) and 0.856 (95% CI = 0.746–0.967) in the training set and validation set, respectively. Compared with the SAPS-II system, the NRI values of the training and validation sets of our model were 0.543 (95% CI = 0.147–0.940) and 0.784 (95% CI = 0.282–1.286), respectively. The IDI values of the training and validation sets were 0.064 (95% CI = 0.004–0.123; *P* = 0.037) and 0.103 (95% CI = 0.002–0.203; *P* = 0.046), respectively. The calibration plot and HL test results confirmed that our model prediction results showed good agreement with the actual results, where the HL test values of the training and validation sets were *P* = 0.8 and *P* = 0.95, respectively. The DCA curve revealed that our model had better clinical net benefit than the SAPS-II system.

**Conclusion:**

We explored the in-hospital mortality of patients with cervical spine fractures without spinal cord injury and constructed a nomogram to predict their prognosis. This could help doctors assess the patient’s status and implement interventions to improve prognosis accordingly.

## Background

Although cervical fractures are rare in trauma patients, their incidence is increasing gradually. A study reported that the incidence of cervical fractures in trauma patients increased from 4.1% in 2005 to 5.4% in 2013. Although the number of cervical spine fractures caused by motor vehicle accidents has decreased significantly, it remains the most common cause of cervical spine fractures, followed by falling from a height [1]. Notably, although the hospitalization expenses due to cervical spine fractures have increased significantly, constituting a major medical care burden, the proportion of hospitalization deaths has not changed significantly during this period [2, 3].

Recent research on patients with cervical spine fractures has primarily focused on prevalence, mortality, and related risk factors in this population; however, these do not fulfill the needs of the clinicians and patients' families regarding the prognosis of the patients [4, 5]. Furthermore, intensive care physicians managing trauma patients mainly focus on chest trauma and hip fractures [6–8]. Insufficient attention to patients with cervical spine fractures often results in poor prognosis. Therefore, we chose this particular population for our research.

Our prediction model is based on routine clinical and laboratory indicators, thus ensuring its ease of implementation in clinical work. Nomograms have been proven effective for conducting clinically personalized risk assessments by integrating potential risk factors; hence, they are widely used to predict the prognosis of specific populations [9, 10]. Utilizing the Medical Information Mart for Intensive Care (MIMIC) III database, this study aimed to establish a prediction model of the in-hospital mortality of patients with cervical spine fractures without spinal cord injury.

## Materials and methods

### Data source

The data used in this retrospective study were obtained from the MIMIC-III database, a large, free-of-charge intensive care database created by the Massachusetts Institute of Technology, containing the data of 53,423 patients admitted to the intensive care unit (ICU) of Beth Israel Deaconess Medical Center (Boston, Massachusetts, USA) between 2001 and 2012 [11]. Since the patient names are hidden in the MIMIC-III database for privacy protection, the need for informed consent was waived in this study. According to the data usage agreement, ZhibinXing completed the training for participant protection in human research (Certificate No.: 48590713) and managed the acquisition and analysis of the research data.

### Study population

**Fig. 1:**
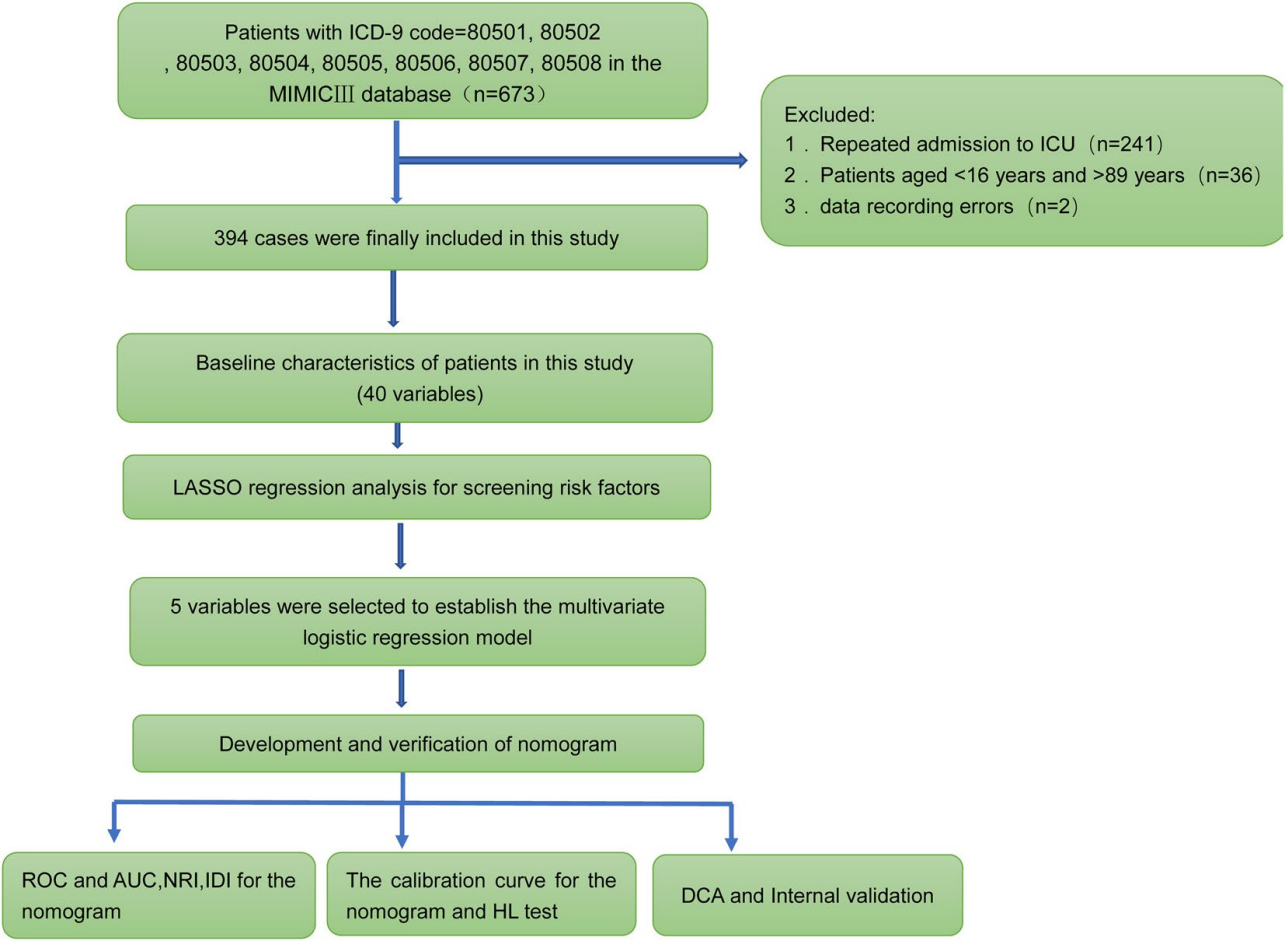
Workflow of the study. ICU, Intensive care unit; MIMIC-III, Medical Information Mart for Intensive Care III; LASSO, Least absolute shrinkage and selection operator; ROC, Receiver operating characteristic; AUC, Area under the receiver operating characteristic curve; NRI, Net reclassification improvement; IDI, Integrated discrimination improvement; HL test, Hosmer‒Lemeshow test; DCA, Decision curve analysis

We used the structured query language in Navicat Premium version 15 to extract the required data. According to the patient's HADM _ ID and ICUSTAY _ ID, we extracted information from the MIMIC-III database for 673 patients with the International Classification of Diseases, ninth edition, codes 80501, 80502, 80503, 80504, 80505, 80506, 80507, and 80508. For patients with multiple ICU admissions, only data from the first ICU admission were retained (*n* = 241). Patients aged < 16 years and > 89 years (*n* = 36) and those with data recording errors (*n* = 2) were excluded. Finally, 394 patients were included in this study (Fig. 1).

### Data extraction

Demographics, vital signs, laboratory tests, comorbidities, and scoring systems data were extracted using the patient's HADM _ ID and ICUSTAY _ ID. The demographic data included sex and age, and the vital signs included heart rate (HR), systolic blood pressure (SBP), diastolic blood pressure (DBP), mean arterial pressure, respiratory rate (RR), and body temperature (T). The laboratory investigation indicators included oxygen saturation (SpO2), glucose, anion gap, bicarbonate, creatinine, chloride, hematocrit, hemoglobin, platelets, potassium, partial thromboplastin time (PTT), international normalized ratio (INR), prothrombin time, sodium, blood urea nitrogen (BUN), white blood cells, calcium, magnesium, red blood cells (RBC), and red blood cell distribution width. The comorbidity indicators were congestive heart failure, cardiac arrhythmias, hypertension, chronic pulmonary, diabetes, renal failure, liver disease, coagulopathy, and fluid electrolyte imbalance. The scoring systems included the Glasgow coma scale (GCS), simplified acute physiology score (SAPS) II, and sequential organ failure assessment score (SOFA).

### Statistical analysis

Missing data are a common problem in research. The treatment of missing data can be roughly divided into three categories: not processing, direct deletion, and filling. In this study, variables with a missing value ratio > 20% were eliminated. For variables with a missing value ratio < 20%, the mice R package in the R language was used for multiple imputations. Applying multivariate imputation by MICE can reduce bias in the feature selection process [12, 13]. Multiple imputation is a mechanism for creating multiple complete datasets in which for each missing value we calculate 5 predictions [14]. We used the predictive mean matching method to generate 5-group complete datasets. Then, the patients were randomly divided into the training set and validation set in a ratio of 7:3. The Shapiro–Wilk test was used to determine whether a continuous variable had a normal distribution. If a continuous variable was normally distributed, it was described as the mean and standard deviation. If a continuous variable was not normally distributed, it was described as the median and interquartile range, and Wilcoxon’s rank-sum test was selected for comparison between two groups. Categorical variables were expressed as frequency/percentage, and the Chi-square test or Fisher’s exact test was used to compare different groups. The Least Absolute Shrinkage and Selection Operator (LASSO) regression method was then used to screen out independent risk factors for mortality. Through cross-validation, we selected the largest λ value with an average error within 1 standard deviation to determine the variables included in the model. A multivariate logistic regression model was established using the selected variables and a nomogram was drawn. Discrimination of the model was assessed using receiver operating characteristics (ROC), area under the ROC curve (AUC), net reclassification improvement (NRI), and integrated discrimination improvement (IDI). The calibration curve and Hosmer–Lemeshow (HL) test were used to reflect the consistency between the actual probability and predicted probability. A decision curve analysis (DCA) was conducted to evaluate the clinical net benefit and clinical applicability of the nomogram. This study was based on the transparent reporting of individual prognosis or diagnosis (TRIPOD) of multivariate predictive models guidelines for analysis and reporting [15].

All statistical analyses were performed using R language (version 4.2.2); A two-sided *P* < 0.05 was considered statistically significant.

## Results

### Baseline characteristics of the patients

This study included 394 patients, who were divided into the survival group (*n* = 352) and the death group (*n* = 42) based on their survival outcome. According to our statistics, the in-hospital mortality rate for patients with cervical spine fractures without spinal cord injury was 10.7%. Compared with the survival group patients, those in the death group were older and had a higher RR, and lower SpO2, SBP, DBP, body temperature. Moreover, the laboratory values for bicarbonate, hemoglobin, and RBC were lower, while those of glucose, anion gap, PTT, INR, and BUN were higher in the death group than in the survival group. The SOFA and SAPS-II scores and the prevalence of fluid electrolyte imbalance were higher in the death group than in the survival group (Table 1).

**Table 1:** Baseline characteristics of in-hospital alive and death groups

Variables	Alive	Death	*p*.overall
	*N* = *352*	*N* = *42*	
Age (years)	52.1 [36.3;72.1]	72.7 [56.8;84.6]	< 0.001
HR (beats/min)	84.8 (15.1)	88.7 (20.4)	0.245
SBP (mmHg)	101 [89.0;111]	81.0 [68.2;96.5]	< 0.001
DBP (mmHg)	47.0 [41.0;55.0]	40.0 [32.2;44.8]	< 0.001
MAP (mmHg)	64.2 [56.0;72.4]	54.5 [38.8;59.0]	< 0.001
RR (beats/min)	16.9 [15.2;19.4]	20.6 [17.5;22.7]	< 0.001
Temperature (°C)	37.1 [36.7;37.4]	36.8 [36.4;37.4]	0.037
SpO2 (%)	94.0 [92.0;97.0]	92.0 [85.0;95.6]	0.003
Glucose (mg/dL)	131 [115;155]	162 [142;185]	< 0.001
Anion gap (mmol/L)	15.0 [13.0;17.0]	17.5 [14.0;20.6]	< 0.001
Bicarbonate (mmol/L)	23.0 [21.0;25.0]	19.0 [17.0;21.0]	< 0.001
Creatinine (mg/dl)	0.90 [0.70;1.10]	1.15 [0.80;1.58]	0.001
Chloride (mmol/L)	104 [101;106]	105 [101;108]	0.175
Hematocrit (%)	38.3 (5.50)	38.4 (6.81)	0.986
Hemoglobin (g/dL)	11.1 [9.50;12.6]	9.35 [8.12;11.2]	< 0.001
Platelets (K/µL)	190 [149;242]	177 [111;209]	0.120
Potassium	3.70 [3.30;4.00]	3.60 [3.32;4.10]	0.820
PTT (s)	26.9 [24.5;31.5]	30.5 [26.2;51.0]	< 0.001
INR	1.20 [1.10;1.30]	1.30 [1.10;1.90]	0.006
PT (s)	13.4 [12.7;14.5]	14.1 [13.0;17.0]	0.006
Sodium (mmol/L)	138 [136;140]	138 [135;140]	0.566
BUN (mg/dL)	16.0 [12.0;21.2]	21.0 [16.0;27.8]	0.001
WBC (K/µL)	14.3 [11.2;18.0]	14.4 [11.6;19.1]	0.203
Calcium (mg/dL)	8.35 [7.90;8.75]	7.88 [7.60;8.60]	0.083
Magnesium (mg/dL)	1.87 [1.70;2.00]	1.87 [1.71;2.00]	0.439
RBC (m/µL)	3.60 (0.70)	3.25 (0.73)	0.005
RDW (%)	13.5 [13.0;14.3]	14.3 [13.5;14.8]	0.006
GCS	15.0 [14.0;15.0]	15.0 [13.2;15.0]	0.442
SAPSII	26.0 [17.0;35.0]	47.0 [36.5;54.8]	< 0.001
SOFA	2.00 [1.00;4.00]	6.00 [3.25;8.00]	< 0.001
Gender, *n* (%)			0.844
Male	228 (64.8%)	26 (61.9%)	
Female	124 (35.2%)	16 (38.1%)	
Congestive heart failure, *n* (%)			0.295
No	315 (89.5%)	35 (83.3%)	
Yes	37 (10.5%)	7 (16.7%)	
Cardiac arrhythmias, *n* (%)			0.627
No	284 (80.7%)	32 (76.2%)	
Yes	68 (19.3%)	10 (23.8%)	
Hypertension, *n* (%)			0.423
No	244 (69.3%)	26 (61.9%)	
Yes	108 (30.7%)	16 (38.1%)	
Chronic pulmonary, *n* (%)			0.193
No	307 (87.2%)	33 (78.6%)	
Yes	45 (12.8%)	9 (21.4%)	
Diabetes, *n* (%)			0.127
No	314 (89.2%)	34 (81.0%)	
Yes	38 (10.8%)	8 (19.0%)	
Renal failure, *n* (%)			1.000
No	336 (95.5%)	41 (97.6%)	
Yes	16 (4.55%)	1 (2.38%)	
Liver disease, *n* (%)			0.441
No	336 (95.5%)	39 (92.9%)	
Yes	16 (4.55%)	3 (7.14%)	
Coagulopathy, *n* (%)			0.113
No	338 (96.0%)	38 (90.5%)	
Yes	14 (3.98%)	4 (9.52%)	
Fluid electrolyte imbalance, *n* (%)			0.011
No	289 (82.1%)	27 (64.3%)	
Yes	63 (17.9%)	15 (35.7%)	

HR, heart rate; SBP, systolic blood pressure; DBP, diastolic blood pressure mean; MAP, mean arterial pressure; RR, respiratory rate; RBC, red blood cell; RDW, red blood cell distribution width; WBC, white blood cell; BUN, blood urea nitrogen; PT, prothrombin time; PTT, partial thromboplastin time; INR, international normalized ratio; GCS, Glasgow Coma Scale; SAPSII, Simplified Acute Physiology Score II; SOFA, sequential organ failure assessment score

### Risk factor screening

**Fig. 2:**
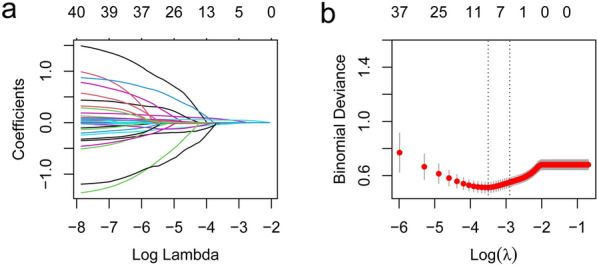
Clinical variables were selected using the least absolute shrinkage and selection operator logistic regression model. **a** Tuning parameter (λ) selection using LASSO penalized logistic regression with fivefold cross-validation. **b** LASSO coefficient profiles of the radiomic features

Based on the LASSO regression analysis and cross-validation, we identified the following 5 of the 40 variables to build a multi-factor logistic regression model (Fig. 2): SBP (odds ratio [OR] = 0.99, 95% confidence interval [CI] = 0.96–1.01), RR (OR = 1.15, 95% CI = 1.05–1.27), SpO2 (OR = 0.98, 95% CI = 0.95–1.00), bicarbonate (OR = 0.92, 95% CI = 0.84–1.01), and SAPS-II (OR = 1.07, 95% CI = 1.04–1.11) (Table 2).

**Table 2:** Multivariate regression model based on LASSO regression results

Variables	Multivariable logistics model		
	Coefficients	OR (95%CI)	*P*-value
SBP	− 0.01418	0.99 (0.96–1.01)	0.2097
RR	0.14303	1.15 (1.05–1.27)	0.0041
SpO2	− 0.02202	0.98 (0.95–1.00)	0.0909
BICARBONATE	− 0.083446	0.92 (0.84–1.01)	0.0816
SAPSII	0.06953	1.07 (1.04–1.11)	< 0.001

SBP, systolic blood pressure; RR, respiratory rate; SAPSII, simplified acute physiology score II

### Development and verification of the nomogram

**Fig. 3:**
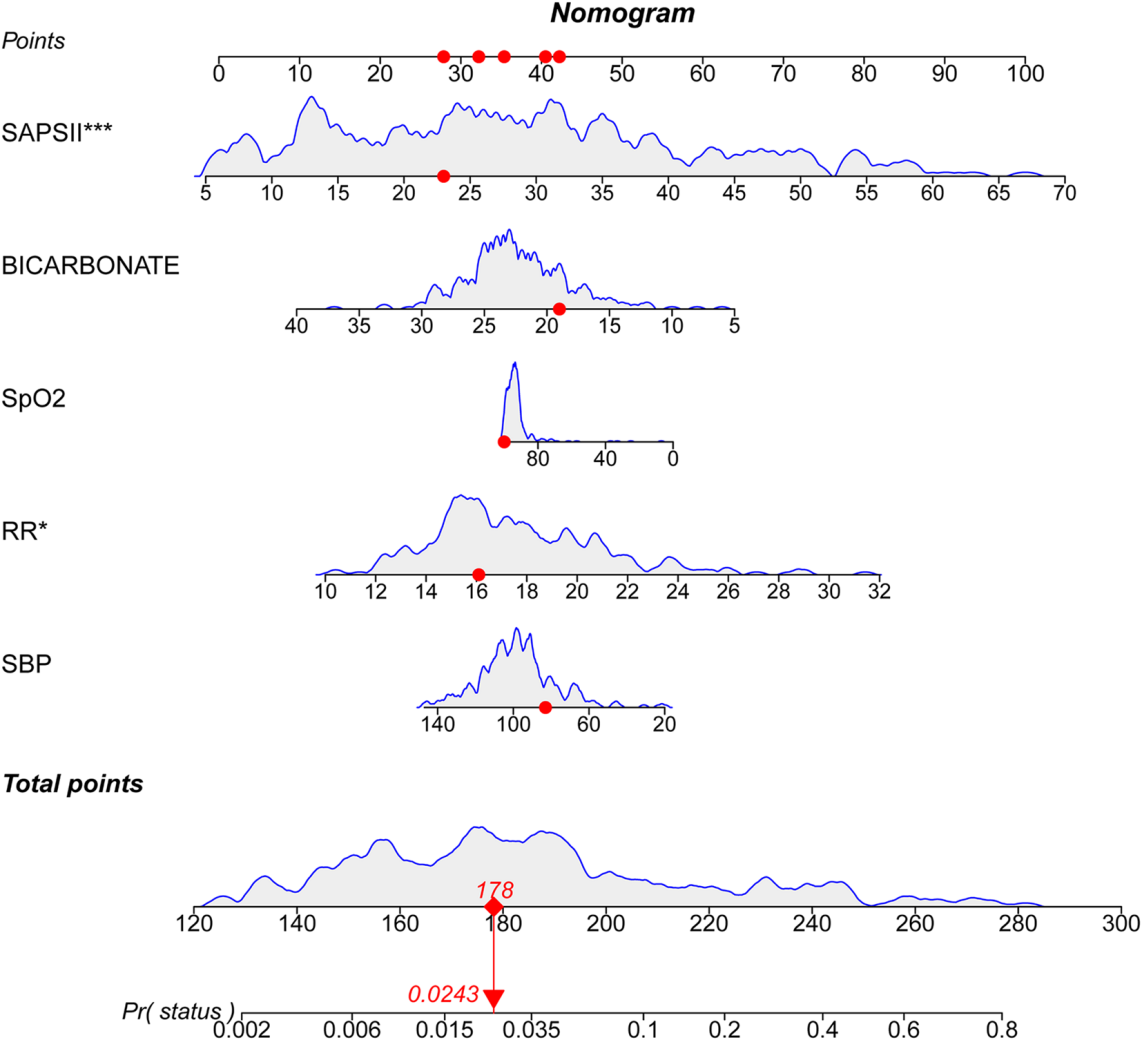
Nomogram for predicting the risk of in-hospital mortality in patients with cervical spine fractures in the ICU. SBP, systolic blood pressure;RR,respiratory rate;SAPSII,simplified acute physiology score II. * means p < 0.05,** means p < 0.01,*** means p < 0.001

**Fig. 4:**
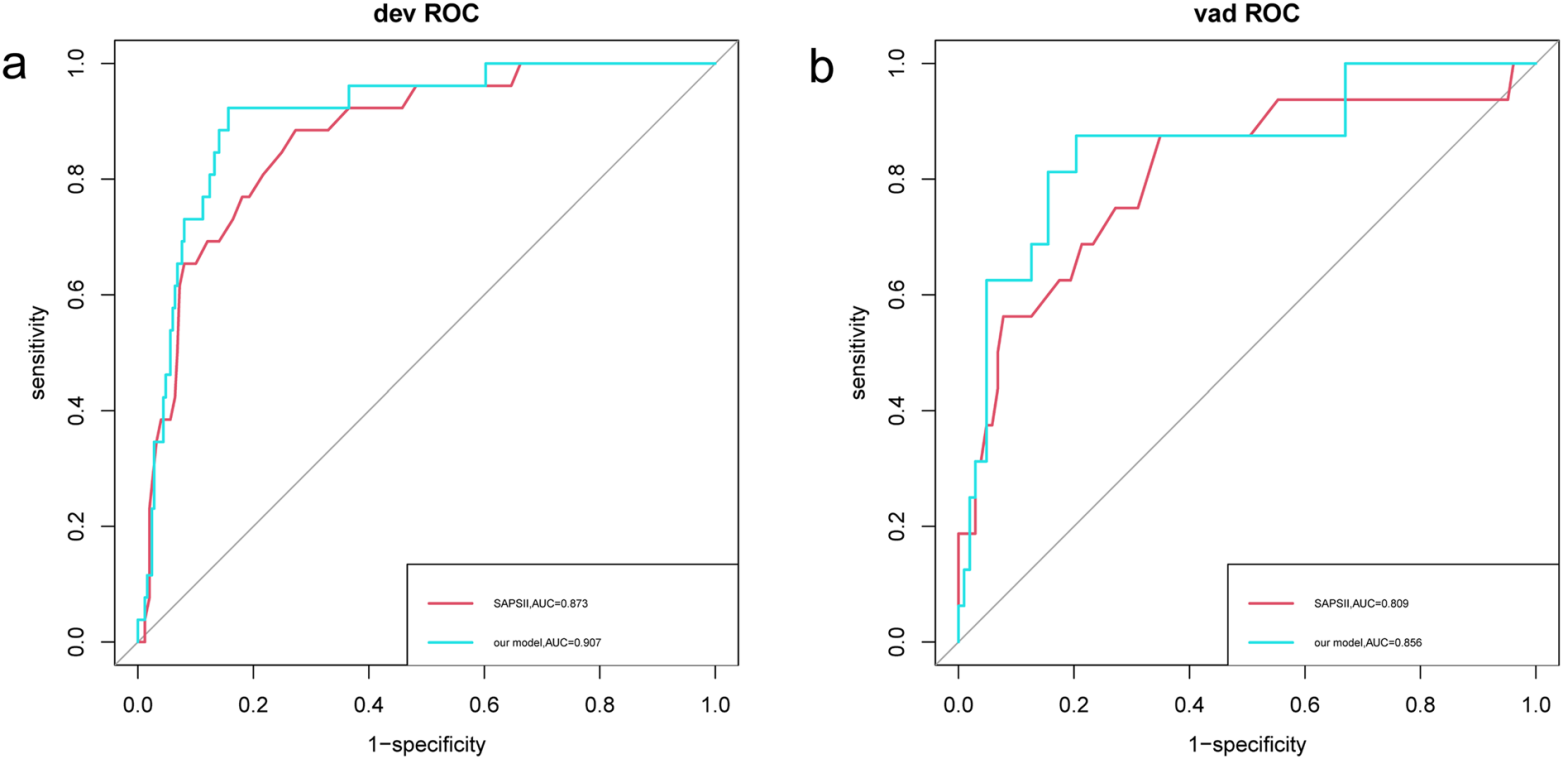
Receiver operating characteristic curve of the established nomogram and SAPSII. **a** Training cohort, **b** Validation cohort. SAPSII, Simplified Acute Physiology Score II; AUC, Area under the receiver operating characteristic curve

**Fig. 5:**
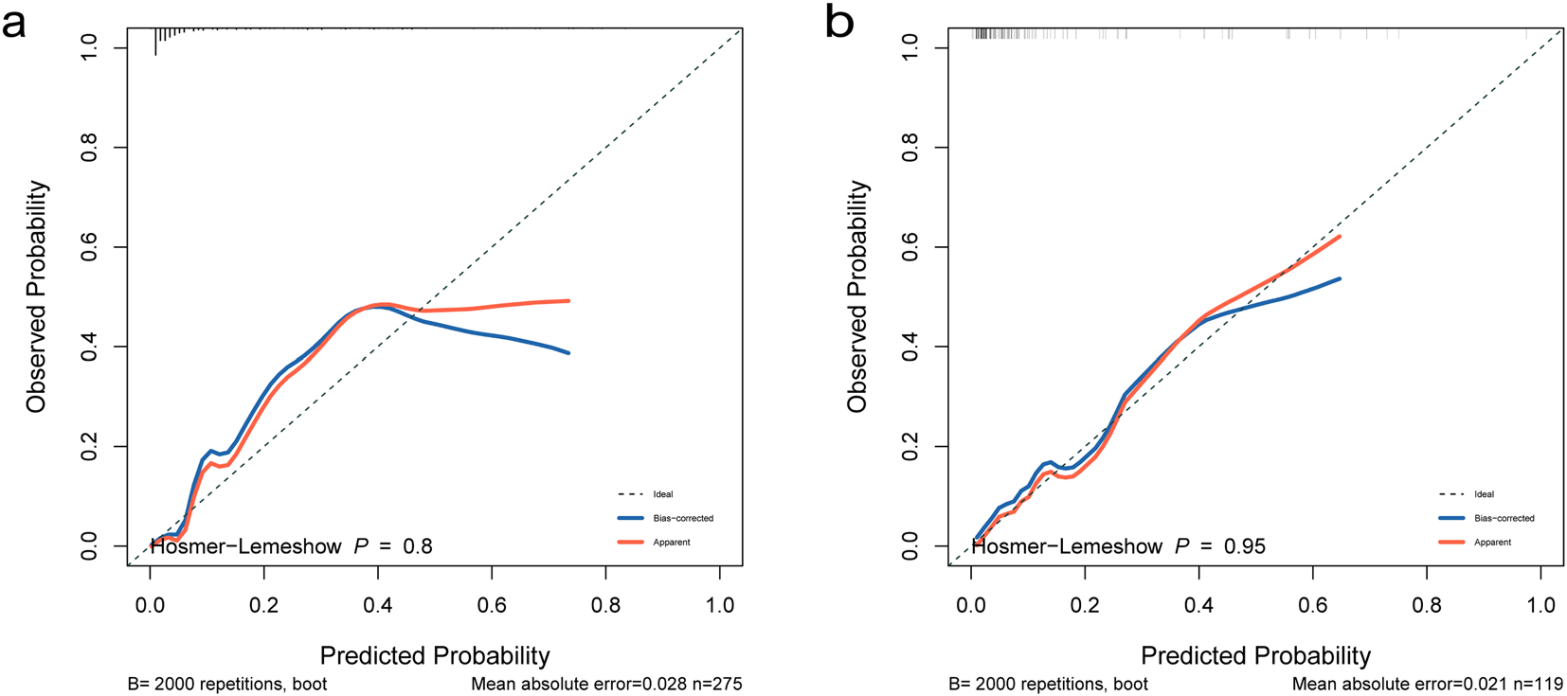
Calibration curve of the established nomogram. **a** Training cohort, **b** Validation cohort

**Fig. 6:**
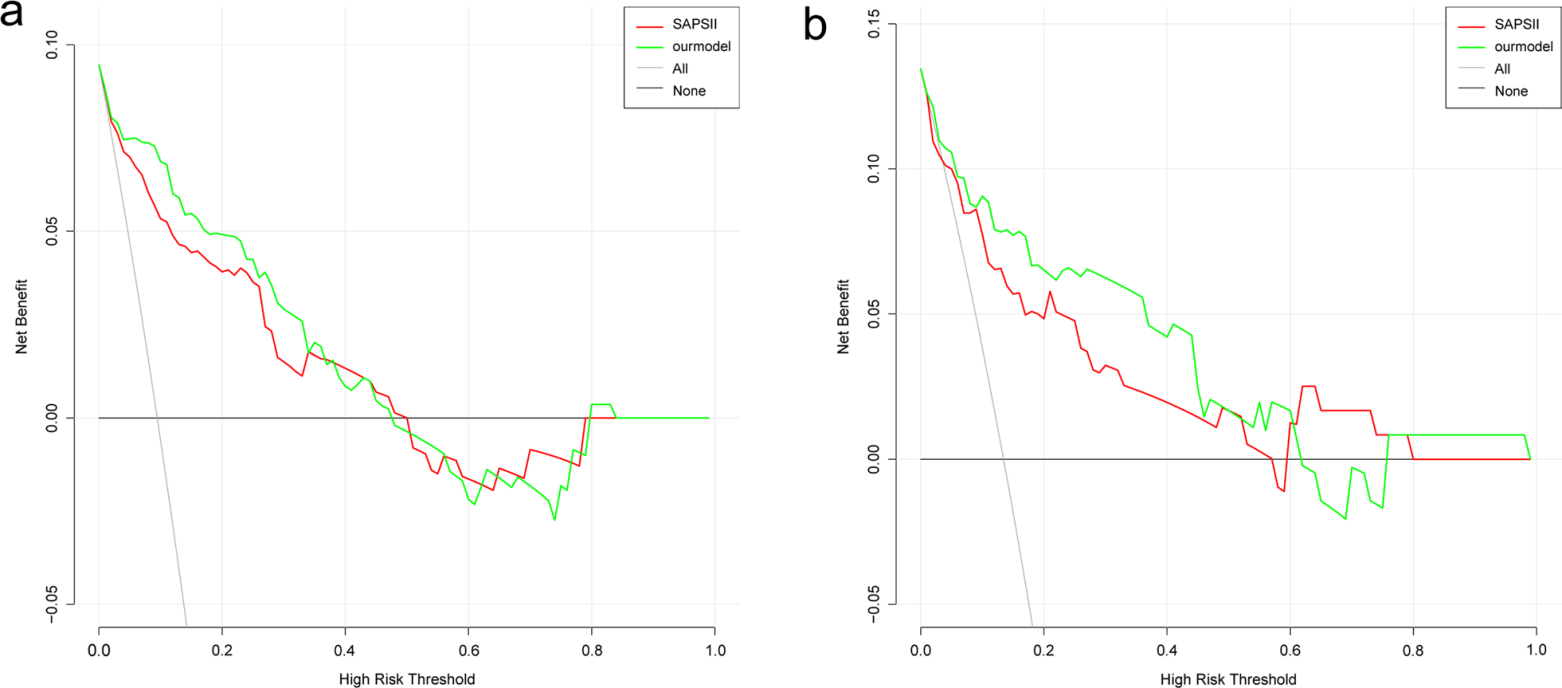
Decision curve analysis of the established nomogram and SAPS II. **a** Training cohort, **b** Validation cohort. SAPSII, Simplified Acute Physiology Score II

Based on the multivariate logistic regression model, we developed a nomogram to predict the in-hospital mortality in patients with closed cervical fractures (Fig. 3). To assess the prediction performance of the nomogram, we drew an ROC curve and calculated the AUC, NRI, and IDI values. The AUC of the training set was 0.907 (95% CI = 0.853–0.961), and that of the verification set was 0.856 (95% CI = 0.746–0.967) (Fig. 4). Compared with the SAPS-II system, the nomogram NRI values of the training and verification sets were 0.543 (95% CI = 0.147–0.940) and 0.784 (95% CI = 0.282–1.286), respectively, and the IDI values were 0.064 (95% CI = 0.004–0.123; *P* = 0.037) and 0.103 (95% CI = 0.002–0.203; *P* = 0.046), respectively. These results showed that our model had good prediction ability. Additionally, the calibration plot and HL test results confirmed that our model prediction results were in good agreement with the actual results, with the HL test value of *P* = 0.8 for the training set and *P* = 0.95 for the validation set (Fig. 5). Finally, the DCA curve illustrated the clinical applicability of the nomogram and compared it with the SAPS-II scoring system (Fig. 6), showing that our model had a greater clinical net benefit than the SAPS-II system.

## Discussion

Recently, the incidence of spinal injuries has increased rapidly. The lumbar spine is the most commonly affected segment, followed by the thoracic spine and cervical spine. Notably, patients with cervical spine injuries experience the highest average length of hospital stay, hospitalization expenses, and mortality rate [16]. Although many studies have described the incidence and mortality rate of cervical spine fractures, few established a specific model to predict the prognosis of the patients. Our study aimed to fill this gap; to the best of our knowledge, this is the first study to predict the in-hospital mortality in patients with cervical spine fractures without spinal cord injury.

In this study, we developed a nomogram comprising five predictors-SBP, SpO2, RR, bicarbonate, and SAPS-II, and performed internal validation of the model. The AUC, NRI, IDI, calibration curve, and HL test results, and other indicators prove that our nomogram exhibits good discrimination and calibration capabilities. This tool could help clinicians in the early identification of high-risk patients, enabling proactive medical interventions to improve patient prognosis.

Metabolic acidosis in major trauma patients has been independently associated with mortality, with severity predictive of outcome in critically ill patients [17]. As shown in Table 1, low bicarbonate level is an independent risk factor for patient mortality. Our study showed that the bicarbonate level in the survival group was 23.0 mmol/L, which was significantly higher than that in the death group (19.0 mmol/L) (*P* < 0.001). Previous studies have found that low serum bicarbonate levels are associated with the progression and increased risk of death in chronic kidney disease [18, 19]. Another study determined that the relationship between bicarbonate and mortality was not related to the pH of the system, indicating that bicarbonate levels were associated with mortality, regardless of whether the potential acid–base disorder was metabolic acidosis or respiratory alkalosis [20]. Although alkaline therapy can improve the prognosis of patients, high serum bicarbonate and metabolic alkalosis should probably be avoided.

SBP serves as an indicator facilitating early detection of hemodynamic abnormalities and reflecting the cardiac output and tissue perfusion. Moreover, it is relatively easy to record the SBP and assess the therapeutic effects. Hypotension can lead to insufficient tissue perfusion and microcirculation disturbance, which in turn leads to organ dysfunction and affects the prognosis of patients. A retrospective study showed that critically ill patients with acute kidney injury having low SBP at admission had an increased risk of in-hospital death as compared to those with normal SBP [21]. Moreover, studies have shown that hypotension is associated with increased in-hospital mortality, myocardial injury, and acute kidney injury in patients with sepsis [22]. In this study, the SBP of the death group was significantly lower than that of the survival group (*P* < 0.001). Thus, relatively high blood pressure is a protective factor in patients with cervical spine fractures, which is consistent with the description in the aforementioned study. Appropriate systolic blood pressure can improve patient outcomes. Current strategies for the treatment of hypotension often include fluid resuscitation, inotropes, and/or vasopressors. A study on the association between elevated systolic blood pressure and the prognosis of critically ill patients showed that all amounts of time at an SBP level between 120 and 140 mmHg in patients after early ICU admission were associated with decreased mortality after ICU admission [21]. Due to the heterogeneity of the etiology and clinical manifestations of patients, management of hypotension can be challenging and multifactorial.

In most patients, peripheral capillary SpO2 has a wide range of consistency with arterial SpO2. Since its measurement is simple and convenient, and it is a non-invasive procedure, it is usually used as an alternative indicator of arterial SpO2 in clinical practice [23–25]. However, studies on the relationship between SpO2 and mortality in trauma patients are limited, and the conclusions are inconsistent. A study reported that SpO2 had no significant effect on the other variables in predicting mortality in patients with severe trauma [26]. However, other studies have suggested that SPO2 can effectively predict early mortality in trauma patients. Compared with the use of each variable alone, their combined use has a better predictive ability for mortality [27, 28]. The reason for the inconsistent results of this study could be that higher blood SpO2 levels are not necessarily associated with better patient survival and too low or too high blood SpO2 levels will have adverse effects on the patients [29].

RR is a readily available clinical indicator for early identification of high-risk patients after trauma, and shortness of breath correlates strongly with 24-h mortality [30, 31]. This study confirmed that RR is a risk factor for death in patients with cervical spine fractures. The SAPS-II scores were significantly higher in the death group than in the survival group (*P* < 0.001), showing a positive correlation with in-hospital mortality. SAPS-II is a predictive tool that is widely used to assess mortality [32], and we integrated this scoring system into our model.

However, our study has some limitations. First, the data were extracted from the MIMIC-III database, which constituted a single-center study; therefore, data from different medical institutions are needed for external verification. Second, due to the design of retrospective studies, potential selection bias is inevitable; hence, the results should be interpreted cautiously. Third, it is obvious that some variables were not included in the model, and they could have a significant effect on the in-hospital mortality of patients with cervical spine fractures.

## Conclusion

Our study found that SBP, SpO2, RR, bicarbonate, and SAPS-II were predictors of in-hospital death in patients with cervical spine fractures without spinal cord injury. We established and validated the logistic regression model and nomogram. Further studies are required to determine whether interventions focused on the nomogram improve clinical prognosis in this population.

## Data Availability

The datasets used and/or analyzed during the current study are available from the corresponding author on reasonable request.
